# Cross-dataset adaptation of voxel-level deep radiomics for predicting survival in inoperable locally advanced NSCLC treated with immunotherapy

**DOI:** 10.3389/fimmu.2026.1787518

**Published:** 2026-03-03

**Authors:** Jin Wang, Zhaoyu Jiang, Wenhao Ji, Han Cheng, Zhen Zhang, Andre Dekker, Leonard Wee, Meng Yan, Xiaojing Lai

**Affiliations:** 1 Department of Radiation Oncology, Zhejiang Cancer Hospital, Hangzhou Institute of Medicine (HIM), Chinese Academy of Sciences, Zhejiang Key Laboratory of Particle Radiotherapy Equipment, Hangzhou, China; 2 School of Public Health, Nanjing Medical University School of Public Health, Nanjing, China; 3 Department of Radiation Oncology (Maastro), GROW Research Institute for Oncology and Reproduction, Maastricht University Medical Centre+, Maastricht, Netherlands; 4 Department of Radiation Oncology, Key Laboratory of Cancer Prevention and Therapy, Tianjin Medical University Cancer Institute & Hospital, National Clinical Research Center for Cancer, Tianjin’s Clinical Research Center for Cancer, Tianjin, China

**Keywords:** deep learning, immunotherapy, lung cancer, overall survival, voxel radiomics

## Abstract

**Background and purpose:**

Predicting overall survival (OS) for inoperable locally advanced non-small cell lung cancer (LA-NSCLC) treated with immune checkpoint inhibitors remains challenging due to heterogeneous clinical response. Furthermore, the application of advanced deep learning is hindered by limited immunotherapy datasets. This study aimed to develop a novel prognostic framework by integrating voxel-level deep radiomics derived from pretreatment imaging with a knowledge transfer strategy to accurately predict OS.

**Materials and methods:**

A total of 526 patients were respectively identified. A non-immunotherapy dataset from the RTOG 0617 clinical trial was used to pre-train a Vision-Mamba deep learning model to learn tumor characteristics within manually delineated tumor regions. Voxel-level radiomics feature maps were generated within tumors and integrated with CT images for dual-input co-training. Using the same dual-input, a cross-dataset transfer learning strategy was then used to adapt the pre-trained models to the immunotherapy context by fine-tuning. The model’s performance was evaluated using the concordance index (C-index), time-dependent area under the receiver operating characteristic curve, Kaplan-Meier survival analysis, calibration curves, and decision curve analysis. Additionally, Gradient-weighted Class Activation Mapping (Grad-CAM) was employed to suggest a possible interpretation of the model’s decision logic.

**Results:**

The proposed model demonstrated robust generalization ability. In the independent immunotherapy testing dataset, the model achieved a C-index of 0.73 (95% CI:0.63-0.82). The time-dependent AUCs for predicting 1-year and 2-year OS were 0.73 and 0.70, respectively. Calibration curves showed good agreement between predicted and observed survival probability. Stratification analysis showed distinct survival differences, with the high-risk group exhibiting significantly poorer OS compared to low-risk group (P<0.001).

**Conclusion:**

We developed a voxel-level deep radiomics framework that bridges the data gap in immunotherapy research through fine-tuning on a limited immunotherapy dataset, and subsequent validation on an independent immunotherapy testing dataset, demonstrating robust generalizability.

## Introduction

Immune checkpoint inhibitors (ICIs) have prolonged survival in patients with inoperable locally advanced non-small cell lung cancer (LA-NSCLC), and have become a standard therapy in current clinical practice guidelines (1, 2). However, clinical outcomes remain highly variable. While some patients experience long-lasting benefits, rapid disease progression is seen in others (3). This difference underscores the urgent need for better pre-treatment prediction of overall survival (OS) to optimize treatment decisions (4). By identifying high-risk patients early, physicians can potentially intensify treatment for those likely to derive little benefit from standard immunotherapy, or alternatively avoiding unnecessary overtreatment in low-risk patients (5).

To address this challenge, radiomics has emerged as a promising, non-invasive tool to analyze tumor phenotypes, predict OS, and guide treatments (6, 7). However, traditional radiomics typically extract hand-crafted features from the entire tumor volume, which implies we cannot spatially resolve phenotypical heterogeneity inside the tumor. A more effective approach turns out to be voxel-level radiomics, which enables us to derive heuristic spatial clusters from feature-by-feature “heatmaps”. While voxel level strategies have been explored in other cancer (8), their potential to uncover the complexity of LA-NSCLC and predict immunotherapy outcomes remains underexplored.

Moreover, implementing advanced deep learning models in the field of immunotherapy faces a challenge due to limited data availability (9). Deep learning models rely on large datasets to learn robust patterns, but immunotherapy has only become widely used in recent years, resulting in relatively small datasets (10). As a result, training complex models on these limited datasets increases the risk of overfitting and poor generalizability. To address this problem, we propose a novel knowledge transfer strategy (11). By leveraging extensive historical data from non-immunotherapy treatments, the Vision-Mamba can be first pre-trained in a supervised manner to learn stable tumor imaging representations, using overall survival as the training endpoint. This pre-training step can capture tumor characteristics that could be transferred to immunotherapy treated cohorts. Such pre-trained features can then be adapted to the specific context of immunotherapy, effectively bridging the data gap between small sample sizes and high-dimensional feature learning (12).

In this study, we developed a novel prognostic framework for inoperable LA-NSCLC by combining voxel-level deep radiomics with a cross-cohort training strategy. Specifically, by leveraging large, high-quality non-immunotherapy datasets for pre-training and subsequently fine-tuning on immunotherapy cohorts, our model was designed to provide accurate OS predictions with high generalizability.

## Methods

### Patient enrollment and study design

**Figure 1:**
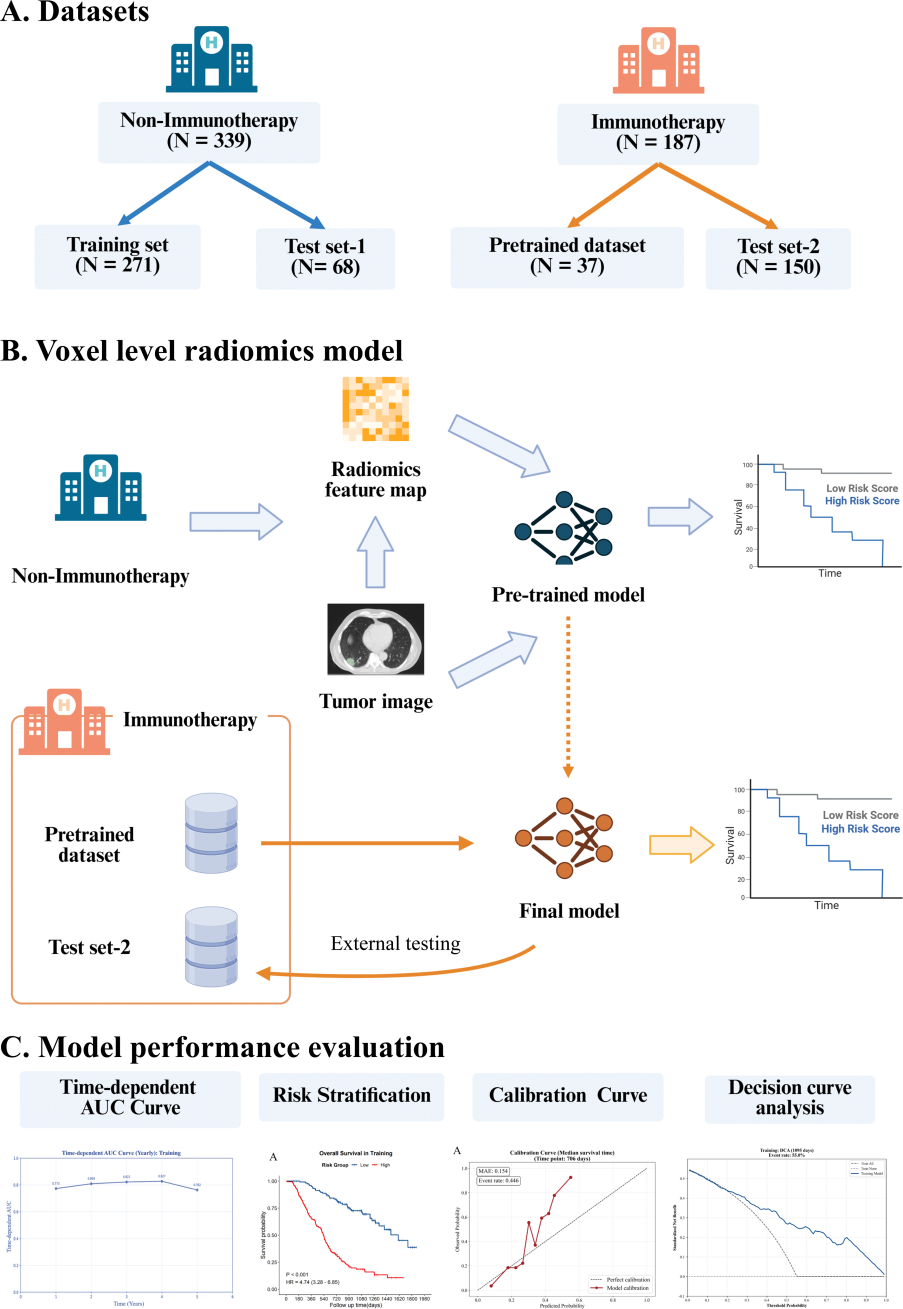
Overall study design and workflow of the voxel-level radiomics deep learning model. **(A)** Data allocation across different treatment groups. **(B)** Voxel level radiomics deep learning model architecture and transfer learning strategy. The deep learning framework employs dual input architecture, simultaneously integrating tumor images and their corresponding voxel level features maps to capture intratumoral heterogeneity. A base model was first developed using the non-immunotherapy dataset. Then we transferred these weights and fun-tuned them on a small immunotherapy dataset, yielding the final model optimized for the immunotherapy dataset. The final model was evaluated on an independent external immunotherapy dataset. **(C)** Performance evaluation. The prognostic performance of the models was comprehensively assessed using time-dependent ROC analysis, Kaplan-Meier risk stratification, calibration plots, and decision curve analysis. AUC, area under the receiver operating characteristic curve.

This retrospective, multi-center study was approved by the Institutional Review Board (reference number: bc20240049), and the requirements for re-acquiring patient informed consent were waived by the Institutional Review Board. As **Figure 1A** illustrated, to implement our knowledge transfer strategy, the patients of the study were divided into group 1 (general chemoradiotherapy) and group 2 (immunotherapy).

We selected in a large cohort of 339 patients treated with chemotherapy without immunotherapy from RTOG 0617 clinical trial, and this dataset was randomly divided into training set and test set at a ratio of 8:2. Detailed inclusion process are shown in **Supplementary Material A**. The immunotherapy dataset consisted of 187 inoperable LA-NSCLC patients treated with immunotherapy and chemoradiotherapy from Tianjin Medical University Cancer Institute & Hospital. To validate the efficacy of our transfer learning strategy, 20% of the patients were randomly allocated to the fine-tuning dataset, while the remaining 80% served as an independent external testing set to evaluate the model’s generalization performance.

The primary endpoint of the study was OS, defined as the time interval from the date of the start of definitive radiotherapy to the date of death from any cause or the last follow-up. Survival status was obtained from routinely maintained institutional clinical databases and follow-up records. Detailed inclusion and exclusion criteria for both datasets are provided in **Supplementary Material A**.

### Image preprocessing and tumor segmentation

Pre-radiotherapy planning computed tomography (CT) images were retrospectively collected and all images were converted from Digital Imaging and Communications in Medicine (DICOM) format to NIfTI (nii) format using the SimpleITK library to enable standardized image processing. Image intensities were normalized using a window setting (window width: 250HU; window level: 30HU), followed by Z-score normalization to ensure consistency. All CT images were resampled to an isotropic voxel size of 1mm*1mm*5mm (linear interpolation). Additional details of imaging acquisition parameters are provided in **Supplementary Material B**.

The primary lung tumor was defined as the region of interest (ROI), as shown in **Figure 1B**. To minimize inter-observer variability, all tumors were independently manually contoured by two junior radiation oncologists. Then, all tumor contours were subsequently reviewed and refined by a senior radiation oncologist with over ten years’ experience in thoracic malignancies. Discrepancies were resolved by consensus discussion. All radiation oncologists were blinded to OS.

### Generation of voxel level radiomics feature maps

Voxel level radiomics feature maps were generated for all axial CT slices containing tumor regions, following a strategy previously described (8). Briefly summarizing here: within the ROI, pixel-wise radiomic features were extracted using a sliding window approach, which allowed local image characteristics to be quantified while preserving spatial heterogeneity across the tumor. For each patient, a total of 90 radiomic features maps were calculated, including 17 intensity features and 73 texture features, which all conformed to the Image Biomarker Standardization Initiative (IBSI) guidelines according to the developers’ documentation webpage. All feature maps were normalized using Z-score normalization and discretized into fixed bins prior to model input. We spatially aligned the radiomics feature maps and stacked them with the corresponding CT slices to form multi-channel input.

To reduce the redundancy and identify informative radiomic representations, each voxel level radiomics feature map was individually evaluated for its predictive ability using Vision-Mamba framework. Specifically, separate models were trained using one radiomics feature map at a time. Feature maps achieving an area under the receiver operating characteristic curve (AUC) greater than 0.6 after convergence was retained (**Supplementary Material C**) for subsequent model construction.

### Deep learning framework with knowledge transfer

The prognostic model was developed using the Vision-Mamba architecture, a state-space model based framework that efficiently captures long range dependencies within image data through structured state-space sequence modeling, as previously introduced by us (8). Its ability to model global contextual relationships with linear computational complexity makes it particularly suitable for analyzing high-resolution voxel-level feature maps, where capturing both local texture and broader spatial patterns is important for characterizing intratumoral heterogeneity. The network took as input multi-channel image representations consisting of pretreatment CT images and tumor voxel-level radiomics feature map. Model training was supervised using time-to-event overall survival data, with censoring information incorporated through a Cox-based survival loss. Consistent with the established pipeline, the network was initialized with convolutional blocks for feature extraction and dimensionality reduction. This was followed by a series of Vision-Mamba encoders responsible for hierarchical feature encoding and long-range dependency modeling. Finally, the final prediction head directly output a continuous survival risk score.

Model training was conducted in two stages using an identical dual-input configuration in both phases, consisting of pretreatment images and voxel-level radiomics feature maps. In the first stage, the entire network was trained using a larger cohort of patients treated without immunotherapy, allowing the model to learn general imaging representations of lung tumors related to OS. In the second stage, the model was fine-tuned using the immunotherapy dataset, without changing the input structure of prediction task. During this phase, the convolutional blocks and the early Vision-Mamba blocks were frozen to preserve generalizable image features, whereas the later Vision-Mamba blocks and the final prediction head were updated to adapt the model to imaging patterns associated with immunotherapy outcomes.

Training was performed using the AdamW optimizer with a batch size of 16, and five-fold cross-validation was applied within the training cohort, the initial learning rate was 1e–4. Data augmentation, including random horizontal flipping, rotation within ±60 degrees, and scaling between 0.8 and 1.2, was applied during training to improve robustness. To enhance interpretability, gradient-weighted class activation mapping (Grad-CAM) was applied to visualize tumor regions that contributed most strongly to the model’s risk prediction.

### Model evaluation and statistical analysis

The predictive performance was evaluated using the time-dependent receiver operating characteristic curves and concordance index (C-index). To ensure the robustness of these metrics, 95% confidence intervals for the C-index were estimated using bootstrap resampling with 1,000 iterations. The agreement between the predicted survival probabilities and the actual observed survival probabilities was assessed using calibration curves. Decision curve analysis (DCA) was conducted to calculate the net benefit. For survival analysis, patients were stratified into high-risk and low-risk groups based on the median risk scores generated by the final model. Patients were stratified into high-risk and low-risk groups based on the median value of the continuous risk scores generated by the final model across the training cohort. Survival distributions were presented using Kaplan-Meier curves and the differences between groups were compared using the Log-rank test.

For patient characteristics, continuous variables were evaluated using the Mann-Whitney U test for comparisons between two groups, or the Kruskal-Wallis (K-W) test for comparisons involving more than two groups. Categorical variables were compared using the Chi-square test or Fisher’s exact test, as appropriate. All statistical tests were two-sided, and a P value less than 0.05 was considered statistically significant.

All statistical analysis were performed using R (version 4.4.1). Deep learning models were constructed and trained using Python (version 3.10.19), implemented within the PyTorch (version 2.1.1) framework. The scikit-learn python library was used for metric calculation and the PyRadiomcs (version 3.1.0) python library using for feature extraction was also integrated into the workflow. All experiments were conducted on an NVIDIA RTX A6000 GPU.

## Results

### Patient characteristics

A total of 526 patients with inoperable LA-NSCLC were included in the study. Baseline clinical characteristics of the four datasets are summarized in **Table 1**. Significant differences were observed between the non-immunotherapy and immunotherapy cohorts.

**Table 1:** Baseline clinical characteristics of the study cohorts.

Characteristics	Overall N = 526	Training set N = 271	Test set-1 N = 68	Fine-tuning dataset N = 37	Test set-2 N = 150	P value
Gender						<0.001*
Female	168 (31.9%)	114 (42.1%)	29 (42.6%)	3 (8.1%)	22 (14.7%)	
Male	358 (68.1%)	157 (57.9%)	39 (57.4%)	34 (91.9%)	128 (85.3%)	
Age (years)	64.0 (57.0, 69.0)	63.0 (56.0, 68.0)	64.0 (56.0, 70.0)	63.0 (57.0, 68.0)	65.0 (59.0, 69.0)	0.448
Pathology						<0.001*
non-SCC	272 (51.7%)	167 (61.6%)	34 (50.0%)	12 (32.4%)	59 (39.3%)	
SCC	254 (48.3%)	104 (38.4%)	34 (50.0%)	25 (67.6%)	91 (60.7%)	
PS Score						0.278
0	316 (60.1%)	164 (60.5%)	36 (52.9%)	25 (67.6%)	91 (60.7%)	
1	208 (39.5%)	107 (39.5%)	32 (47.1%)	12 (32.4%)	57 (38.0%)	
2	2 (0.4%)	0 (0.0%)	0 (0.0%)	0 (0.0%)	2 (1.3%)	
Stage						<0.001*
IIB	15 (2.9%)	0 (0.0%)	0 (0.0%)	4 (10.8%)	11 (7.3%)	
IIIA	305 (58.0%)	184 (67.9%)	49 (72.1%)	11 (29.7%)	61 (40.7%)	
IIIB	180 (34.2%)	87 (32.1%)	19 (27.9%)	15 (40.5%)	59 (39.3%)	
IIIC	26 (4.9%)	0 (0.0%)	0 (0.0%)	7 (18.9%)	19 (12.7%)	
cT						0.952
1	21 (11.2%)	\	\	4 (10.8%)	17 (11.3%)	
2	75 (40.1%)	\	\	15 (40.5%)	60 (40.0%)	
3	40 (21.4%)	\	\	9 (24.3%)	31 (20.7%)	
4	51 (27.3%)	\	\	9 (24.3%)	42 (28.0%)	
cN						0.169
0	12 (6.4%)	\	\	0 (0.0%)	12 (8.0%)	
1	23 (12.3%)	\	\	7 (18.9%)	16 (10.7%)	
2	99 (52.9%)	\	\	18 (48.6%)	81 (54.0%)	
3	53 (28.3%)	\	\	12 (32.4%)	41 (27.3%)	
Radiotherapy Technique						<0.001*
3D-CRT	191 (36.3%)	150 (55.4%)	41 (60.3%)	0 (0.0%)	0 (0.0%)	
IMRT	268 (51.0%)	121 (44.6%)	27 (39.7%)	20 (54.1%)	100 (66.7%)	
VMAT	67 (12.7%)	0 (0.0%)	0 (0.0%)	17 (45.9%)	50 (33.3%)	
Smoking Status						<0.001*
No	87 (16.5%)	18 (6.6%)	5 (7.4%)	9 (24.3%)	55 (36.7%)	
Yes	423 (80.4%)	241 (88.9%)	59 (86.8%)	28 (75.7%)	95 (63.3%)	
Unknown	16 (3.0%)	12 (4.4%)	4 (5.9%)	0 (0.0%)	0 (0.0%)	
Induction chemotherapy						0.850
No	26 (13.9%)	\	\	6 (16.2%)	20 (13.3%)	
Yes	161 (86.1%)	\	\	31 (83.8%)	130 (86.7%)	
Unknown	339	271	68	0	0	
Concurrent chemotherapy						<0.001*
No	106 (20.2%)	0 (0.0%)	0 (0.0%)	26 (70.3%)	80 (53.3%)	
Yes	420 (79.8%)	271 (100.0%)	68 (100.0%)	11 (29.7%)	70 (46.7%)	
Consolidation chemotherapy						<0.001*
No	159 (30.2%)	31 (11.4%)	9 (13.2%)	29 (78.4%)	90 (60.0%)	
Yes	367 (69.8%)	240 (88.6%)	59 (86.8%)	8 (21.6%)	60 (40.0%)	
Cetuximab						0.787
No	182 (53.7%)	144 (53.1%)	38 (55.9%)	\	\	
Yes	157 (46.3%)	127 (46.9%)	30 (44.1%)	\	\	

Data is presented as number (%) for categorical variables and median (interquartile range) for continuous variables. Differences among four datasets were assessed using the Kruskal-Wallis test for continuous variables and Pearson’s x^2^ test or Fisher’s exact test for categorical variables.

The “\” categories were excluded from the statistical analysis.

The “*” means P value < 0.05.

3D-CRT, three-dimensional conformal radiation therapy; IMRT, intensity-modulated radiation therapy; PS, performance status; SCC, squamous cell carcinoma; VMAT, volumetric modulated arc therapy.

Compared with the non-immunotherapy training and testing cohorts, the immunotherapy cohort, drawn from a Chinese population, included more male patients and squamous cell carcinoma and was characterized by different radiotherapy techniques. Specifically, intensity modulated radiotherapy and volumetric modulated arc therapy were predominantly used in immunotherapy, whereas three-dimensional conformal radiotherapy was mainly applied in the non-immunotherapy cohort. In addition, the immunotherapy testing cohort exhibited a smaller planning target volume compared with the no-immunotherapy testing cohort. A direct comparison of clinical characteristics between the immunotherapy testing cohort and the non-immunotherapy testing cohort is provided in **Supplementary Table 1**.

### Robustness and generalizability of the knowledge transfer learning model

**Figure 2:**
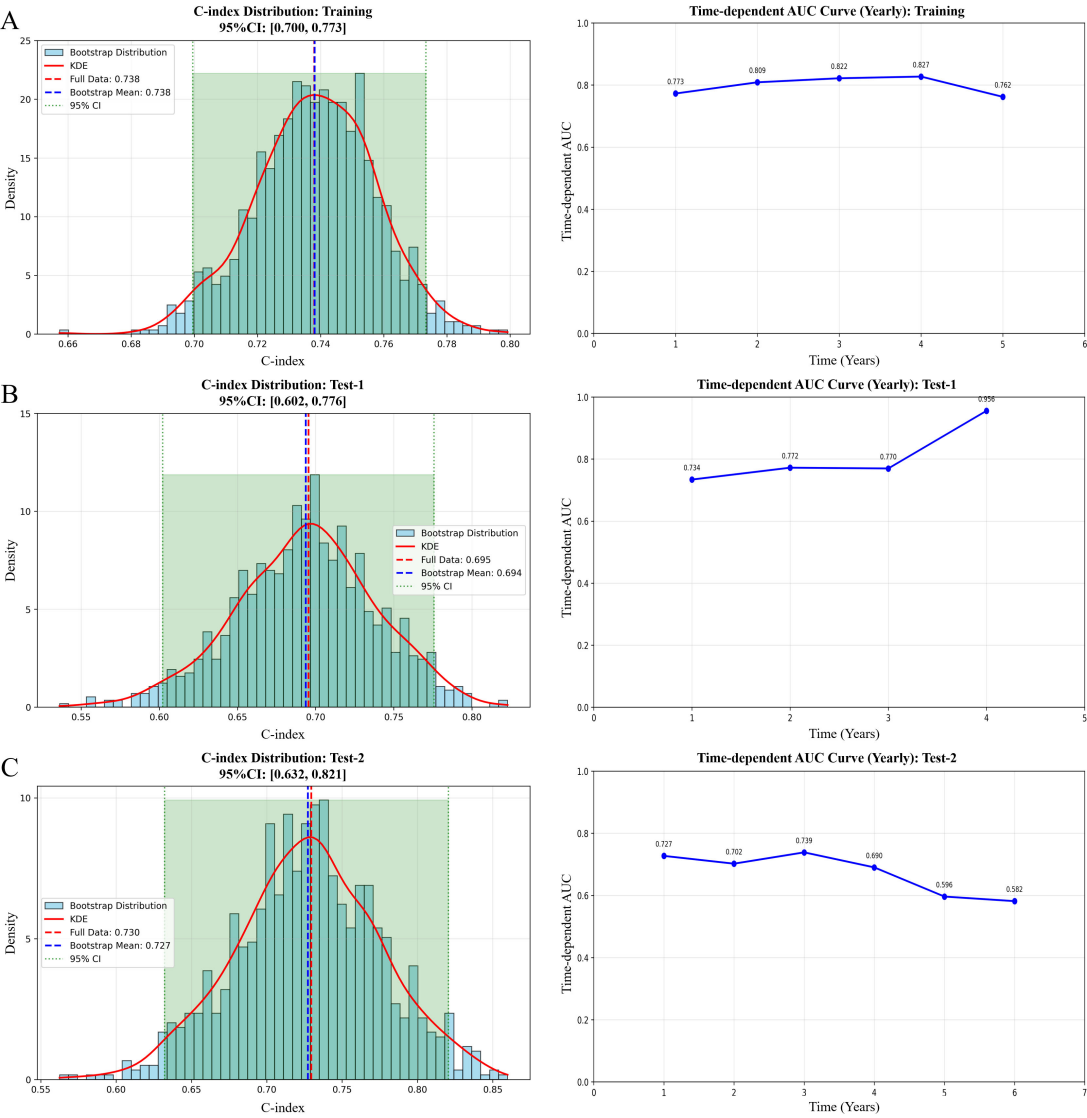
Performance evaluation of the models. The evaluation is presented for **(A)** the training set, **(B)** the test set-1, **(C)** the test set-2 (the independent immunotherapy testing cohort). C-index, concordance index; KDE, kernel density estimation; CI, confidence interval; AUC, area under the receiver operating characteristic curve.

Model performance was evaluated using time-dependent receive operating characteristic analysis and the C-index across the training cohort, test set-1 (non-immunotherapy cohort) and test set-2 (immunotherapy cohort). In the training cohort, the model achieved a C-index of 0.74, and the time-dependent AUCs remained stable across the evaluated time points (**Figure 2A**). When applied to the non-immunotherapy testing cohort, the model yielded a C-index of 0.69 (**Figure 2B**).

Model performance in the independent immunotherapy testing cohort is shown in **Figure 2C**. In this test set, the model demonstrated consistent discrimination, with 1-year and 2-year AUC values of 0.73 and 0.70, respectively. The C-index reached 0.73 (95% CI: 0.63-0.82), as summarized in **Table 2**. Time-dependent AUCs remained relatively stable during early follow-up and gradually declined at later time points. The performance comparison across the training set, test set-1 and test set-2 is summarized in **Supplementary Table 3**.

**Table 2:** Predictive performance of the voxel level radiomics model across study cohorts.

Metric	Training set	Test set-1	Test set-2 (Immunotherapy)
C-index [95% CI]	0.738 [0.700-0.773]	0.694 [0.602-0.776]	0.727 [0.632-0.821]
1-year AUC	0.773	0.734	0.727
2-year AUC	0.809	0.772	0.702
3-year AUC	0.822	0.770	0.739

C-index, concordance index; CI, confidence intervals; AUC, area under the receiver operating characteristic curve.

**Figure 3:**
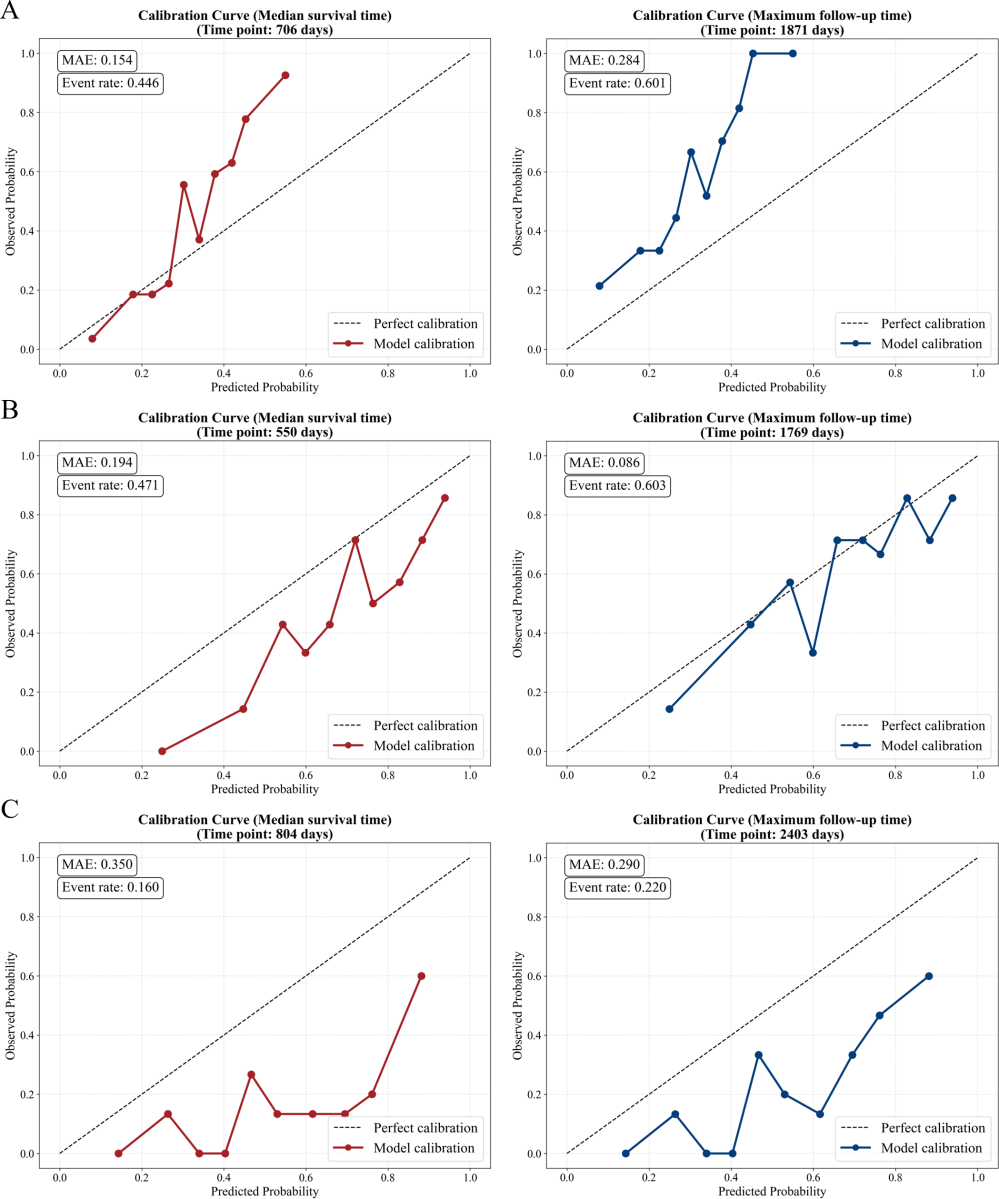
Calibration curve of the models across different datasets. The calibration performance is evaluated for **(A)** the training set, **(B)** the test set-1, **(C)** the test set-2 (the independent immunotherapy testing cohort). MAE, mean absolute error.

Calibration curves across the training set, test set-1 and test set-2 are shown in **Figure 3**. Overall, the calibration curves showed acceptable agreement with the diagonal reference line at both the median survival time and the maximum follow-up time.

### Prognostic stratification and survival outcomes

**Figure 4:**
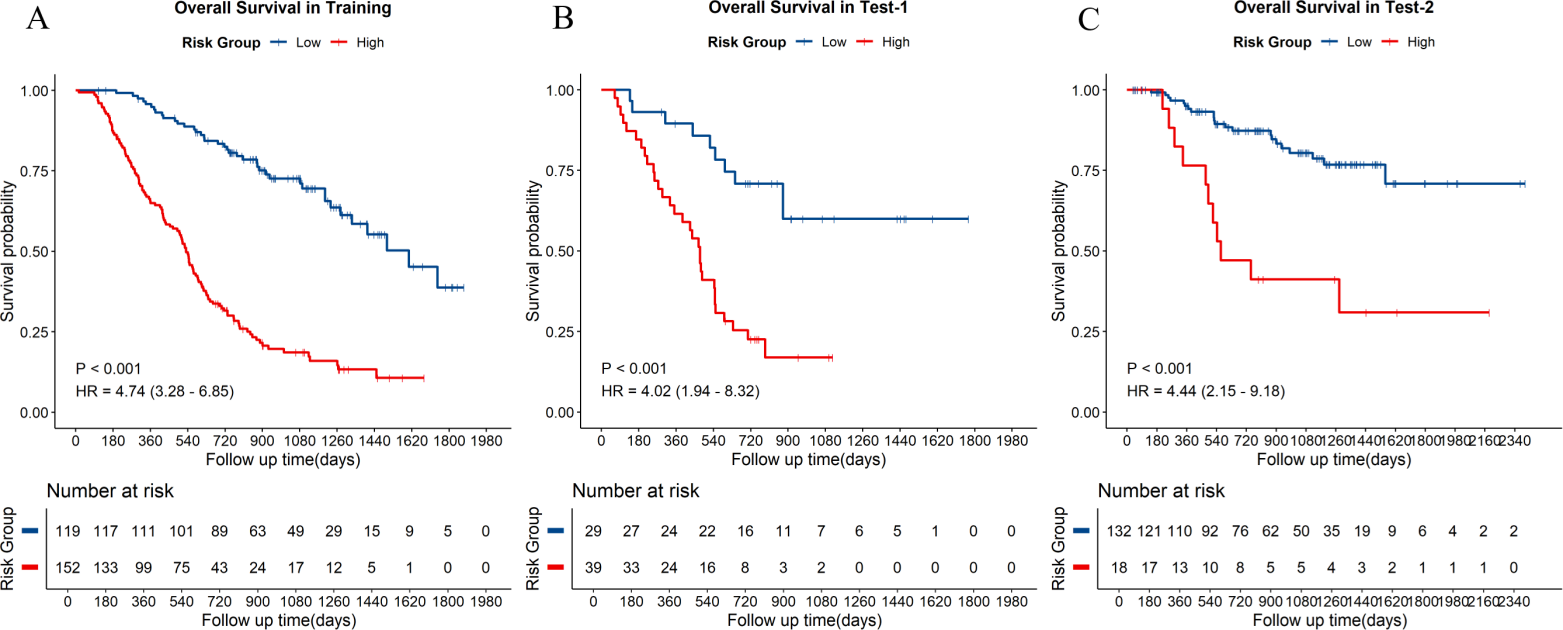
Kaplan-Meier survival estimates risk stratification across datasets. Overall survivals are stratified by the models into low-risk and high-risk groups in **(A)** the training set, **(B)** the test set-1, **(C)** the test set-2 (the independent immunotherapy testing cohort). HR, hazard ratio; CI, confidence interval.

Distinct survival differences between the low-risk and high-risk groups were consistently observed across the training set, the non-immunotherapy testing set, and the independent immunotherapy test set, as illustrated by the Kaplan-Meier curves in **Figure 4**. In all three datasets, patients classified as high risk exhibited significantly poorer overall survival compared with those in the low-risk group (all log-rank P < 0.001). The estimated hazard ratios were 4.74,4.02, and 4.44 in the training set, test set-1 and test set-2, respectively.

### Clinical utility and interpretability analysis

In the training set and test set-1, the model yielded a higher net benefit than both the “treat-all” and “treat-none” across a range of threshold probabilities. In the immunotherapy test cohort, positive net benefit was observed within a more limited threshold range, while the model performance remained above the reference strategies (**Supplementary Figure 1**).

Model interpretability was visualized through two approaches. The voxel-level radiomics feature maps (**Supplementary Figure 2**) illustrated the spatial distribution of textural heterogeneity, confirming that the extraction process successfully captured distinct biological signals invisible on CT. The Grad-CAM analysis (**Supplementary Figure 3**) highlighted the specific sub-regions driving the risk prediction. In patients classified as high risk, attention maps predominantly localized within the tumor region and exhibited spatial gradients, with lower attention weights observed toward the tumor margins. In contrast, low-risk patients showed a more diffuse and relatively uniform distribution of attention within the tumor region.

## Discussion

In the study, we developed and validated a voxel-level deep radiomics framework to predict OS in patients with inoperable LA-NSCLC treated with immune checkpoint inhibitors. By leveraging tumor imaging representations learned from large-scale non-immunotherapy cohorts, and transferring these representations through selective fine-tuning of the Vision-Mamba architecture, the model effectively adapted knowledge encoded in deep imaging features to the immunotherapy setting. The stable discrimination and acceptable calibration observed in the independent immunotherapy testing set indicate that pretreatment CT imaging contains prognostic information that remains informative across different settings and supports the feasibility of applying imaging-based survival models in the immunotherapy era.

Our findings are consistent with prior radiomics and deep learning-based studies in NSCLC, which have reported associations between pretreatment imaging features and OS, including in immunotherapy treated populations (7, 13–16). However, most existing radiomics studies extract features at the whole tumor level, averaging spatial information across the tumor, or were developed and evaluated within relatively small immunotherapy dataset (10, 17). While these approaches have shown encouraging performance, their ability to generalize across evolving clinical settings remains uncertain (18). In contrast, our study focused on preserving intratumoral spatial information and explicitly evaluates generalization in an independent immunotherapy cohort, extending existing work toward more clinically heterogeneous populations. Existing clinical risk scores for LA-NSCLC, predominantly based on factors such as AJCC stage and performance status, provide a foundational prognostic framework. Our voxel-level deep radiomics model adds a complementary, imaging-derived dimension that quantifies intratumoral spatial heterogeneity. This may offer improved risk discrimination and pave the way for more integrated prognostic tools in the immunotherapy era.

The differences observed between the non-immunotherapy and immunotherapy datasets reflect the evolving clinical landscape of inoperable LA-NSCLC (**Table 1**; **Supplementary Table 1**). Changes in radiotherapy techniques and target volume are consistent with shifts in treatment strategies and patient selection accompanying the adoption of immunotherapy and therefore represent inherent heterogeneity in contemporary clinical practice (19, 20). Such heterogeneity poses a challenge for prediction of overall survival models developed within a single treatment context, as treatment patterns may limit generalizability. The preserved performance of our model in the immunotherapy testing dataset suggests that learning generalized imaging representations from historical data, followed by adaptation to patients treated with immunotherapy, is a reasonable method to address this issue. These findings underscore the need for modeling strategies that explicitly account for temporal and therapeutic heterogeneity when developing imaging based prognostic tools for modern oncology practice (21, 22).

The value of voxel-level image representation is highlighted by the model’s ability to capture spatially heterogeneous activation patterns within tumors, as visualized by voxel-level feature maps. Unlike global descriptors, voxel-level features preserve local spatial patterns (**Supplementary Figure 2**). Biologically, high intratumoral heterogeneity is increasingly recognized as a key driver of immune resistance and poor survival (23–25). By decoding these complex spatial phenotypes, our model leveraged spatial complexity for prognostic estimation. This implicit representation may contribute to coherent risk estimation across diverse patients.

Despite the high dimensionality inherent to voxel level radiomics and the limited size of the immunotherapy dataset, the final model achieved a C-index of 0.73 (**Table 2**), alongside acceptable calibration across time points (**Figure 3**). Such stability would be difficult to achieve if voxel level models were trained exclusively on small immunotherapy dataset, where overfitting and unstable risk estimation are common (22). These findings indicate that learning generalized imaging representations from large non-immunotherapy datasets before adaptation is not merely optional but necessary to fully leverage voxel level information in data-limited immunotherapy settings (26).

Regarding the feature selection process, the threshold of AUC > 0.6 was chosen empirically as a baseline filter to retain features with modest univariate prognostic signal while maintaining a manageable subset for downstream modeling. This selection was performed strictly within the training cohort using five fold cross-validation to avoid data leakage. We acknowledge that alternative thresholds or multivariate selection methods could be explored in future work to further optimize the balance between feature informativeness and model complexity.

The gradual decline in time-dependent AUC observed in the immunotherapy testing dataset with increasing time (**Figure 2**) is also clinically plausible. As follow-up extends, survival outcomes are increasingly influenced not only by post-treatment factors that are not captured by baseline imaging, such as subsequent lines of therapy and comorbid conditions, but also by potential biases related to outcome ascertainment during long-term follow-up (27, 28). In addition, the number of patients at risk decreases over time due to censoring and events, which can lead to greater variability in performance estimates. These factors suggest that baseline imaging-based models are inherently better suited for early survival prediction, and that declining long term discrimination reflects the growing impact of dynamic clinical variables, such as subsequent lines of therapy, rather than a limitation of the modeling method itself (29).

From a clinical perspective, the proposed model provides a practical tool for pre-treatment risk stratification in patients with inoperable LA-NSCLC receiving immunotherapy. The ability to identify patients at higher risk of poor survival before treatment initiation may support more individualized treatment planning, such as consideration of intensified multimodal strategies or closer post treatment surveillance (30), while sparing low risk patients from unnecessary escalation (28, 31). For example, a patient classified as high-risk by our model could be prioritized for enrollment in clinical trials exploring intensified immunotherapy regimens (e.g., combination with anti-angiogenic agents or novel immune modulators). Alternatively, such patients might benefit from intensified surveillance protocols, including more frequent follow-up imaging (e.g., CT scans every 2–3 months) to enable early detection of disease progression and prompt intervention. These actionable strategies exemplify how our model could translate into tailored clinical management. To facilitate the practical use of the model in multidisciplinary discussions, its continuous risk score could be translated into categorical tiers (e.g., Low/Intermediate/High) based on clinically meaningful survival probability thresholds, and visualized alongside the CT scans using color-coded scales and attention heatmaps. These actionable strategies exemplify how our model could translate into tailored clinical management. Future efforts should focus on prospective validation, integration with clinical imaging systems, and realworld evaluation of the model’s impact on clinical decision making and patient outcomes.

Several limitations of the study should be acknowledged. First, this study is retrospective in nature. We plan to conduct prospective trials to evaluate the model’s performance in real-world clinical settings, which will be essential for establishing its clinical utility and guiding potential implementation. Second, the use of 2D axial slice optimizes computational efficiency but discards 3D information. Third, variability in CT scanning parameters (e.g., slice thickness, reconstruction kernel) across institutions could affect the stability of radiomic features and the model’s generalizability in real-world practice. Fourth, patient comorbidities, variations in supportive care, and subsequent lines of therapy after progression, were not fully captured in our datasets and may act as unmeasured confounders affecting the model’s predictions and generalizability. Integrating temporal dynamic image features, and dosiomics may further enhance the predictive performance. Finally, key predictive biomarkers relevant to immunotherapy response, such as programmed death-ligand 1 (PD−L1) expression and tumor mutational burden (TMB), were not available in our retrospective cohorts and therefore could not be integrated into the model. Future studies that incorporate such molecular data alongside deep radiomic features hold promise for building more comprehensive and biologically informed prognostic tools. Although voxel-level features improved prognostic performance, the direct biological correlates of these spatially resolved imaging patterns remain incompletely understood. Future studies that integrate voxel-level radiomics with spatially mapped genomic or histopathological data are needed to establish clearer links between these imaging phenotypes and underlying tumor biology, which would enhance the model’s biological plausibility and clinical acceptability.

## Conclusion

In conclusion, we present a robust prognostic framework for inoperable LA-NSCLC that combined voxel level radiomics with transfer learning. By effectively leveraging historical data to characterize intratumoral heterogeneity, the model provides a generalized, non-invasive tool for predicting overall survival in the immunotherapy era, paving the way for more personalized therapy. Future work should focus on prospective multi−center validation, integration with complementary biomarkers, and addressing practical implementation barriers to facilitate clinical translation.

## Data Availability

Training set and test set-1 are available at TCIA (http://doi.org/10.7937/TCIA.2018.jze75u7v) (32, 33). Test set-2 is available from the corresponding author upon reasonable request and institutional ethical approval.
