# Correction: Cross-dataset adaptation of voxel-level deep radiomics for predicting survival in inoperable locally advanced NSCLC treated with immunotherapy

**DOI:** 10.3389/fimmu.2026.1842651

**Published:** 2026-04-17

**Authors:** Jin Wang, Zhaoyu Jiang, Wenhao Ji, Han Cheng, Zhen Zhang, Andre Dekker, Leonard Wee, Meng Yan, Xiaojing Lai

**Affiliations:** 1 Department of Radiation Oncology, Zhejiang Cancer Hospital, Hangzhou Institute of Medicine (HIM), Chinese Academy of Sciences, Zhejiang Key Laboratory of Particle Radiotherapy Equipment, Hangzhou, China; 2 School of Public Health, Nanjing Medical University School of Public Health, Nanjing, China; 3 Department of Radiation Oncology (Maastro), GROW Research Institute for Oncology and Reproduction, Maastricht University Medical Centre+, Maastricht, Netherlands; 4 Department of Radiation Oncology, Key Laboratory of Cancer Prevention and Therapy, Tianjin Medical University Cancer Institute & Hospital, National Clinical Research Center for Cancer, Tianjin’s Clinical Research Center for Cancer, Tianjin, China

**Keywords:** deep learning, Immunotherapy, lung cancer, overall survival, Voxel radiomics

In the **Acknowledgements**, “the mandatory BioRender copyright attribution” was erroneously omitted. The following sentence has been added to the end of the **Acknowledgements**: “**Figure 1** was created using BioRender (Created in BioRender. Zhang, Z. (2026) https://BioRender.com/sgbtr1q; Agreement No. YJ29J52QGU).”.

The original version of this article has been updated.

