# Supplementary material for: Cross-dataset adaptation of voxel-level deep radiomics for predicting survival in inoperable locally advanced NSCLC treated with immunotherapy
Source: Front Immunol. 2026 Mar 3;17:1787518. doi: 10.3389/fimmu.2026.1787518 (PMC12992003; doi:10.3389/fimmu.2026.1787518)
Supplement: Supplementary file 1 [file Table1.docx]

# Supplementary Materials

## Patient enrollment and patient characteristics

The eligibility criteria were as follows: (1) histologically confirmed non-small cell lung cancer (NSCLC); (2) age ≥ 18 years and Karnofsky Performance Status (KPS) ≥ 70; (3) inoperable, locally advanced NSCLC (clinical stage II-III, AJCC 8th edition); (4) receiving definitive chest radiotherapy and immunotherapy. Exclusion criteria across the cohorts included: (1) prior lung surgery before definitive radiotherapy; (2) prior thoracic radiotherapy; (3) low-quality planning CT images insufficient for analysis; (4) unacceptable radiotherapy dose deviation or incomplete treatment; (4) lack of follow-up data.

RTOG 0617 (Clinical trial cohort): Among 544 participants in the RTOG 0617 clinical trial with available CT images, patients were excluded due to poor CT quality and unacceptable dose deviation. The resulting 339 patients received standard-dose (60Gy, N = 187) were included.

**Supplementary Table 1. Comparison of clinical characteristics between the non-immunotherapy (Test set-1) and immunotherapy (Test set-2) cohorts.**

| **Characteristics** | **Test set-1**  **(non-immunotherapy) N = 68** | **Test set-2 (immunotherapy) N = 150** | **P value** |
| --- | --- | --- | --- |
| **Gender** |  |  | **<0.001** |
| Female | 29 (42.6%) | 22 (14.7%) |  |
| Male | 39 (57.4%) | 128 (85.3%) |  |
| **Age (years)** | 64.0 (56.0, 70.0) | 65.0 (59.0, 69.0) | 0.529 |
| **Pathology** |  |  | 0.184 |
| non-SCC | 34 (50.0%) | 59 (39.3%) |  |
| SCC | 34 (50.0%) | 91 (60.7%) |  |
| **Performance status** |  |  | 0.312 |
| 0 | 36 (52.9%) | 91 (60.7%) |  |
| 1 | 32 (47.1%) | 57 (38.0%) |  |
| 2 | 0 (0.0%) | 2 (1.3%) |  |
| **Stage** |  |  | **<0.001** |
| IIB | 0 (0.0%) | 11 (7.3%) |  |
| IIIA | 49 (72.1%) | 61 (40.7%) |  |
| IIIB | 19 (27.9%) | 59 (39.3%) |  |
| IIIC | 0 (0.0%) | 19 (12.7%) |  |
| **Radiotherapy technique** |  |  | **<0.001** |
| 3D-CRT | 41 (60.3%) | 0 (0.0%) |  |
| IMRT | 27 (39.7%) | 100 (66.7%) |  |
| VMAT | 0 (0.0%) | 50 (33.3%) |  |
| **Smoking Status** |  |  | **<0.001** |
| No | 5 (7.4%) | 55 (36.7%) |  |
| Yes | 59 (86.8%) | 95 (63.3%) |  |
| Unknow | 4 (5.9%) | 0 (0.0%) |  |
| **Concurrent chemotherapy** |  |  | **<0.001** |
| No | 0 (0.0%) | 80 (53.3%) |  |
| Yes | 68 (100.0%) | 70 (46.7%) |  |
| **Consolidation chemotherapy** |  |  | **<0.001** |
| No | 9 (13.2%) | 90 (60.0%) |  |
| Yes | 59 (86.8%) | 60 (40.0%) |  |
| **PTV volume (cc)** | 508.6 (371.3, 702.1) | 344.0 (239.9, 450.6) | **<0.001** |

Note: Data is presented as number (%) for categorical variables and median (interquartile range) for continuous variables. P values were calculated using the Chi-square test or Fishier’s exact test for categorical variables, and the Mann-Whitney U test for variables.

*Abbreviations: 3D-CRT, three-dimensional conformal radiation therapy; IMRT, intensity-modulated radiation therapy; SCC, squamous cell carcinoma; VMAT, volumetric modulated arc therapy; PTV, planning target volume.*

## Imaging scanning parameters

For RTOG 0617, the CT scanning parameters are publicly available in their dataset. For test set-2, intravenous contrast-enhanced planning CT scans were acquired on a single Brilliant (Philips Medical Systems; Best, The Netherlands) multislice scanner with a standardized protocol: 120 kVp, 100 mAs, 3 mm slice thickness, 512 x 512 image matrix, 50 cm fields of view, 0.977 mm pixel spacing and vendor’s default convolution kernel. These parameters were consistent with the imaging protocols commonly used in clinical practice.

## Selected radiomic features

**Supplementary Table 2. Summary of the selected radiomics features.**

| **Features** | **Definition** |
| --- | --- |
| original_glszm_LargeAreaHighGrayLevelEmphasis | It quantifies the joint distribution of large size zones and high gray-level values within the region of interest. |
| original_glszm_SizeZoneNonUniformityNormalized | It measures the variability of zone sizes throughout the image. |
| original_ngtdm_Contrast | It quantifies the local intensity variation between a voxel and its surrounding neighbors, captures spatial heterogeneity and texture coarseness. |
| original_firstorder_90Percentile | The high percentile (90th) of the intensity distribution in low-frequency components. |
| original_ngtdm_Coarseness | The coarseness of an image’s texture. |

## Model performance comparison

**Supplementary Table 3. Model performance comparison.**

|  | **Training set** | **Test set-1** | **Test set-2** |
| --- | --- | --- | --- |
| **Vision mamba** |  |  |  |
| C-index [95% CI] | 0.738 [0.700-0.773] | 0.694 [0.602-0.776] | 0.727 [0.632-0.821] |
| **Cox regression** |  |  |  |
| C-index [95% CI] | 0.647 [0.602-0.687] | 0.619 [0.528-0.716] | 0.636 [0.537-0.733] |
| **ResNet18** |  |  |  |
| C-index [95% CI] | 0.726 [0.690-0.761] | 0.692 [0.604-0.769] | 0.659 [0.559-0.761] |

## Decision curve analysis


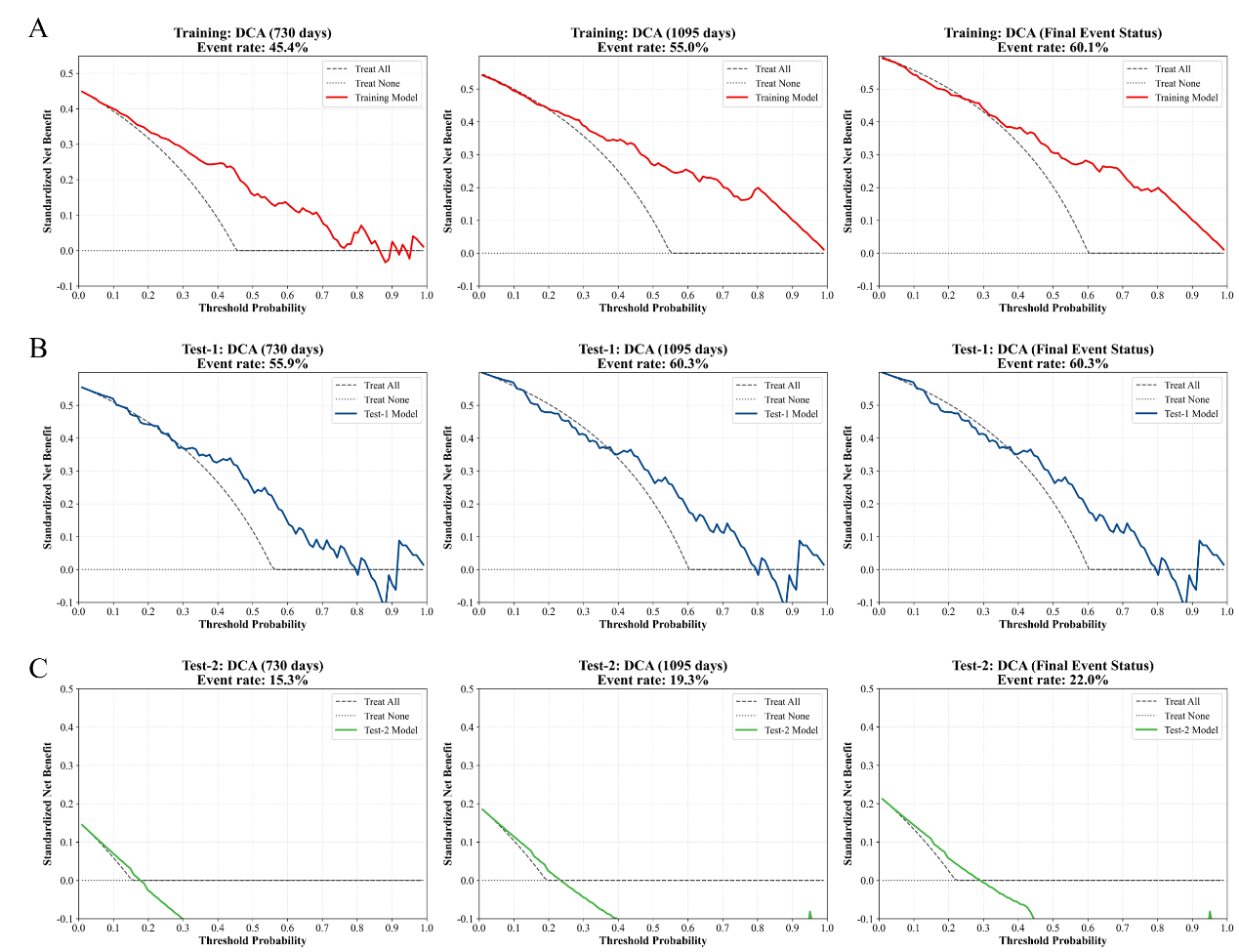


**Supplementary Figure 1. Decision curve analysis of the prognostic model.**

Decision curve analysis was performed in the training set (A), test set-1 (B) and test set-2 (C) at different time points. *Abbreviations: DCA, decision curve analysis.*

## Feature maps


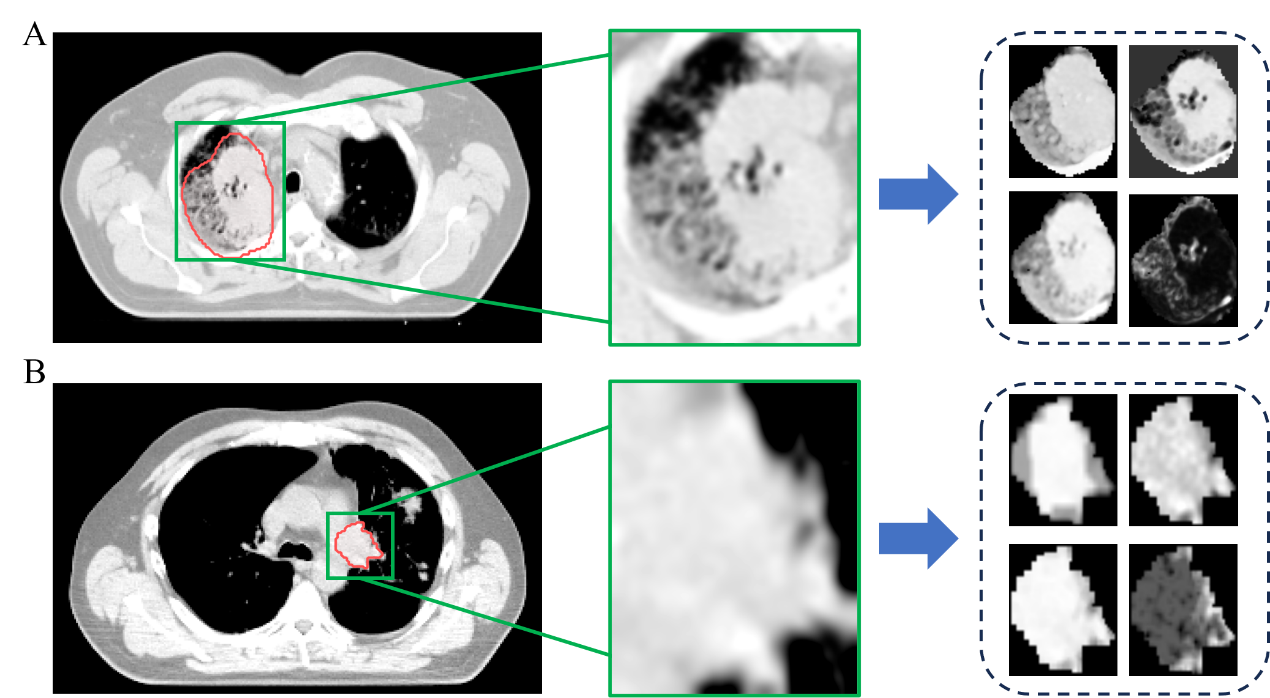


**Supplementary Figure 2. Visualization of voxel-level deep radiomics feature maps.** (A, B) Representative examples of feature extraction results from two different patients.

## Model attention


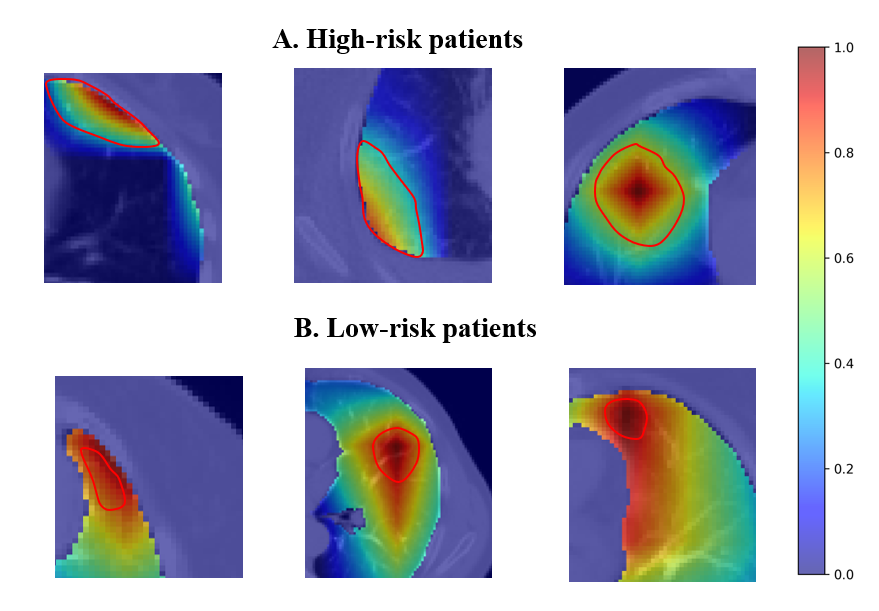


**Supplementary Figure 3. Visualization of model attention using Grad-CAM.**

Representative Grad-CAM heatmaps are shown for patients classified as high-risk (A) and low-risk (B). Color intensity indicates the relative contribution of local image regions to the mode’s survival risk prediction. The red contours represent the overlaid tumor boundaries, corresponding to the spatially aligned tumor contours across slices. In high-risk patients, attention was predominantly concentrated within the tumor region and exhibited a graded distribution, whereas in low-risk patients, attention appeared more diffusely distributed across the tumor areas.
